# Uncoupling Different Characteristics of the *C. elegans* E Lineage from Differentiation of Intestinal Markers

**DOI:** 10.1371/journal.pone.0106309

**Published:** 2014-09-02

**Authors:** Scott M. Robertson, Jessica Medina, Rueyling Lin

**Affiliations:** Department of Molecular Biology, University of Texas Southwestern Medical Center, Dallas, Texas, United States of America; University of North Carolina at Chapel Hill, United States of America

## Abstract

In the 4-cell *C. elegans* embryo, a signal from P_2_ to its anterior sister, EMS, specifies the posterior daughter of EMS, E, as the sole founder cell for intestine. The P_2_-to-EMS signal restricts high level zygotic expression of the redundant GATA transcription factors, END-1 and END-3, to only the E lineage. Expression of END-1 or END-3 in early blastomeres is sufficient to drive intestinal differentiation. We show here that a number of E lineage characteristics, which are also regulated through P_2_-EMS signaling, can be uncoupled from intestine development, and each with a different sensitivity to specific perturbations of the P_2_-EMS signal. For example, we show that the extended cell cycle in Ea and Ep depends on the P_2_-induced high level expression of the cell cycle regulator, WEE-1.1, in E. A mutation in *wee-1.1* results in shortened Ea and Ep cell cycles, but has no effect upon intestinal differentiation or embryogenesis. Furthermore, it has been shown previously that the total number of E lineage cell divisions is regulated by a mechanism dependent upon E being specified as the intestinal founder cell. We now show, however, that cell division counting can be uncoupled from intestine differentiation in the E lineage. Many mutations in P_2_-EMS signal genes exhibit nonfully-penetrant defects in intestinal differentiation. When embryos with those mutations generate intestinal cells, they often make too many intestinal cells. In addition, at the level of individual embryos, expression of *end-1* and *end-3*, and another very early E-specific zygotic gene, *sdz-23*, exhibit stochastic and discordant defects in P_2_-EMS signaling mutants. We show here that *sdz-23* is expressed close to wildtype levels in embryos deleted of both *end-1* and *end-3*. *sdz-23* does not appear to function in intestine development, raising the intriguing possibility that the P_2_-EMS interaction has downstream molecular consequences within the E lineage independent of *end-1/3* and intestinal development.

## Introduction

By the 12-cell stage of *C. elegans* embryogenesis, six founder cells (AB, MS, E, C, D, and P4) have been generated [1]. The lineal descendants of each founder cell exhibit related developmental fates and similar cell cycle timing. Generation of the founder cells appears to be primarily under the control of maternal factors (proteins and mRNAs) deposited into the oocyte prior to fertilization, many of which are uniquely specialized for founder specification [2]. After maternal specification of the founder blastomeres, elaboration of the founder cell developmental program then comes under zygotic control [3], [4].

The progeny of the 8-cell stage E blastomere is a clone of 20 cells that constitute the entire intestine of the animal [1]. Two GATA factors, END-1 and END-3, are expressed zygotically in the E blastomere and function somewhat redundantly for intestinal cell specification [5]–[8]. Loss-of-function mutation in both *end-1* and *end-3* results in an E blastomere that does not produce intestinal cells, and instead produces muscle and epithelial cells. Overexpression of either *end-1* or *end-3* by itself in the early embryo is sufficient to induce non-intestinal precursor cells to express intestinal markers through a molecular cascade similar to that observed in the wildtype E lineage [6], [9]

E is the posterior daughter of the EMS blastomere, which is itself specified by the maternally supplied transcription factor SKN-1 [10]. SKN-1 activates zygotic transcription of nearly identical GATA factors *med-1* and *med-2*, which regulate lineage-specific zygotic transcription in both E and its sister cell MS [11]–[14]. MED-1/2, along with SKN-1, activates transcription of *end-3*, and, to a much lesser extent, *end-1*. In *skn-1* mutants, MS-derived tissues are not produced in 100% of the embryos, whereas only ∼40% of the embryos lack intestinal cells (termed the gutless phenotype), indicating a parallel pathway regulating intestine formation. Contact between EMS and its posterior neighbor, P_2_, is required for E to make intestinal cells [15]. In the absence of the P_2_-EMS interaction, E produces tissue types normally produced by a wildtype MS [16]. Genetic screens have identified components of the Wnt, MAPK and SRC signaling pathways involved in the P_2_-EMS interaction [17]–[22]. These signaling pathways converge on the downstream Wnt effector, POP-1, the sole *C. elegans* TCF protein. We have shown that transcription of the *end-1* gene is directly activated by POP-1/TCF in the E nucleus in response to the P_2_-EMS interaction [23]. The P_2_-EMS signaling pathways restrict transcriptional activation by POP-1, along with the POP-1 coactivator β-catenin, SYS-1, to the E blastomere and promote POP-1-dependent high expression levels of both *end-1* and *end-3* [24], [25]. In MS, POP-1/TCF functions as a repressor of the *end-1* and *end-3* genes [23], [26]. When the P_2_-EMS signal is absent, POP-1 functions as a repressor of *end-1* and *end-3* in both MS and E [23], [26].

One unique characteristic of the E lineage program is that Ea and Ep, the two daughters of E, exhibit an extended cell cycle, compared to the corresponding MS daughters, MSa and MSp (approximately 40–42 minutes versus 22–24 minutes, respectively) [1], [27]. Another defining characteristic of the E lineage is that Ea and Ep, at the 26-cell stage, are the first cells to ingress into the embryo interior at the initiation of gastrulation [1]. The E blastomere also generates a uniquely asymmetric cell division lineage: Ea and Ep undergo three successive rounds of division to generate 16 cells, of which twelve stop dividing while four go on to divide one more time to generate the final 20 intestinal cells. These 20 intestinal cells form a bilaterally symmetric tube with each cell exhibiting a common apical-basolateral polarity with respect to the central lumen [1], [28]. In this study, we will refer to the developmental stage of embryos according to the number of cells derived from a particular founder cell, eg. 2E, 4E, 8AB, etc.

If an E blastomere is isolated and cultured in media, it continues to divide, differentiate, and express multiple intestinal differentiation markers [15], [16], [29], [30]. In addition, the isolated E blastomere exhibits many of the defining characteristics of the E lineage in intact embryos, including the division timing of its descendants, the total number of divisions it undergoes, and certain aspects of cell polarity [15], [28]. Morphogenesis of intestinal cells into a bilaterally symmetric tube does not occur when E differentiates in isolation, as this requires contact with surrounding non-intestinal cells [31]. However, all E descendants in culture media form a cyst and exhibit a common apical-basolateral polarity with respect to the central cavity of the cyst [28]. Most of these characteristics of the E lineage are tightly associated with the ability of E to generate differentiated intestinal cells. In mutant embryos that fail to make intestinal cells, E daughters tend to divide earlier than E daughters in wildtype embryos, and they do not initiate gastrulation. In embryos mutant for *skn-1*, *med-1/2*, and/or *end-1/3*, if intestinal cells are produced, their number is usually abnormal (primarily less than the wildtype 20, but in some cases more) [6], [13]. In embryos carrying a loss-of-function mutation in *cki-1*, a negative regulator of cell cycle progression, or a gain-of-function mutation in *cdc-25*, a positive regulator, the E lineage undergoes one extra round of cell division [32]–[35]. When either of these mutations is combined with a mutation where the E blastomere adopts the fate of MS, the transformed E blastomere undergoes the number of cell divisions and with cell cycle timing typical for a wildtype MS blastomere [35].

The above observations suggest that the P_2_-EMS signal at the 4-cell stage leads to the many different characteristics of the E lineage (E and all of its lineage-restricted descendants), in addition to intestinal development. We know very little about how these characteristics of E lineage fate, other than intestinal differentiation, is regulated by the P_2_-EMS signal. The P_2_-EMS interaction could lead to the direct activation of a master gene, which then regulates a number of downstream target genes, resulting in the generation of the complete E phenotype as outlined above. Alternatively, the P_2_-EMS signals could result in the direct activation of a suite of genes, each of which regulates separate pathways that together lead to the full E lineage phenotype. We and others have identified, through molecular approaches, several zygotically-expressed genes whose restricted or highly-enriched expression in the E blastomere is dependent on the same set of genes that specify E as the intestinal precursor [36], [37]. This includes *sdz-23*, which encodes a protein with an EGF-like motif, and *sdz-26*, which encodes an F-box containing protein. Neither of these genes appears to have a function in the specification of E as an intestinal precursor or in the differentiation of intestinal cells. In fact, no specific function has yet been ascribed to either gene. Identification of these zygotic genes expressed early in the E lineage suggests that the P_2_-EMS interaction has molecular consequences beyond specifying E as the endoderm precursor.

Here, we show that the different characteristics of the E lineage fate can be uncoupled in certain mutants. Many embryos carrying mutations for P_2_-EMS signaling genes can generate an intestinal cell lineage which loses count of the normal number of cell divisions. Mutation in the cell cycle regulator *wee-1.1* results in a shortened cell cycle for E daughters Ea and Ep, without affecting intestinal differentiation or embryogenesis, and these worms are viable. In addition, expression of three E-specific genes, assayed in individual embryos mutant for P_2_-EMS signaling components, are affected discordantly and stochastically. Our results support the model whereby certain aspects of the E lineage program are independently regulated by the P_2_-EMS signaling.

## Results

### Discordance between expression of two E-specific genes and differentiation of gut granules in P_2_-EMS signaling mutants

Many mutations defective in P_2_-EMS signaling have a non-penetrant defect in intestinal formation [19]–[22]. That is, only a certain percentage of mutant embryos fail to produce intestinal cells. Differentiation of intestinal cells is readily scored in live embryos by the generation of intestinal-specific gut granules, which exhibit birefringence under polarized light. We asked whether a correlation exists between the expression of two E-specific genes and the differentiation of gut granules in mutants defective in P_2_-EMS signaling. We used integrated reporters expressing nuclear GFP under the transcriptional control of upstream regulatory sequence from the *end-1* and *sdz-23* genes [23], [37]. Embryos carrying mutations in *mom-1(Porcupine)*, *mom-3(Wntless)*, *mom-4(MAP3K)*, and *mom-5(Frizzled)* were analyzed.

**Figure 1:**
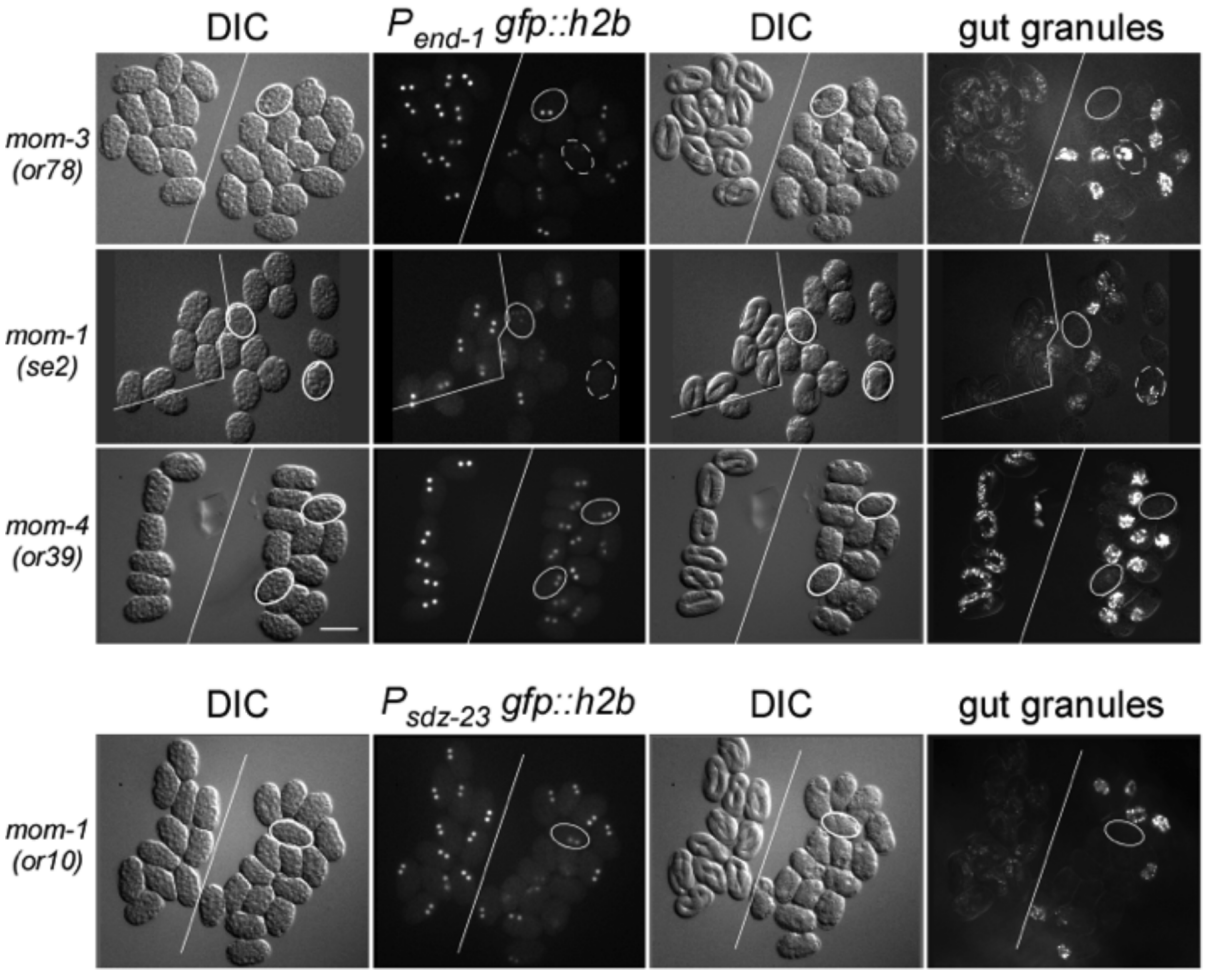
E-lineage reporter expression and intestine development. In each panel, two groups of identically-staged wildtype and mutant embryos (left and right, respectively; divided by solid white line) expressing the same E-specific reporter were mounted together on a slide at around the 8-cell stage. Embryos were imaged for DIC and GFP fluorescence at the 2E stage (left 2 panels), and then imaged again 13 hours later for DIC and birefringent gut granules (right two panels). The mutation scored for each row is indicated on the left. The E-lineage reporter is indicated above the GFP fluorescence panels. Note that all *mom-4(or39)* mutant embryos express the P*end-1*:*gfp*::*h2b* reporter, albeit at lower levels than in wildtype embryos. Solid outline: examples of embryos that are GFP positive at the 2E stage but do not develop gut granules. Dashed outline: examples of embryos that did not express the reporter at the 2E stage but nonetheless went on to generate gut granules. Gut granule birefringence can appear stronger in mutants because of failed morphogenesis leading to compaction of intestinal cells, compared to the fully elongated intestine in wildtype embryos. Bar: 50 µm.

Groups of carefully-staged wildtype and mutant embryos were placed side-by-side and imaged for nuclear GFP fluorescence as a measure of reporter expression. Those same sets of embryos were allowed to develop to a later stage and then scored for birefringent gut granules. Figure 1 shows representative expression of the *end-1* reporter in *mom-1(se2)*, *mom-3(or78)*, and *mom-4(or39)* mutant embryos, as well as representative expression of the *sdz-23* reporter in *mom-1(or10)* mutant embryos. Complete reporter and gut granule formation results are summarized in Table 1. In the wildtype background, both reporters were expressed in all embryos, and all embryos went on to exhibit gut granule birefringence. However, in various mutant backgrounds defective in the P_2_-EMS signal, while there is a strong correlation between embryos lacking gut granule production and those exhibiting abolished or strongly reduced expression of *end-1* or *sdz-23*, discordance was also observed. Selected embryos that are GFP positive but gut granule negative, or GFP negative and gut granule positive, are outlined in Figure. 1. Of the 99 mutant embryos that expressed the *end-1* reporter, and the 39 mutant embryos that expressed the *sdz-23* reporter, 27 (26%) and 19 (49%), respectively, did not develop gut granules. Three of 117 embryos and one of 22 embryos that went on to develop gut granules did not exhibit detectable levels of the *end-1* and *sdz-23* reporters, respectively. The discordance was especially noticeable for *mom-4(ne19)* and *mom4(or39)* embryos, where both of these mutations generate approximately 40% of embryos lacking gut granules, although all express detectable, albeit reduced levels, of both reporters. These results demonstrate the lack of a strict causality between expression of these two early E-lineage restricted genes and intestinal differentiation (as scored by gut granule production) in mutants defective in P_2_-EMS signaling.

**Table 1:** Coexpression of gut reporter GFP and intestinal granules in embryos defective in P_2_-EMS signaling.

mutation	*end-1* reporter				*sdz-23* reporter				combined			
	GFP+		GFP-		GFP+		GFP-		GFP+		GFP-	
	GG+	GG-	GG+	GG-	GG+	GG-	GG+	GG-	GG+	GG-	GG+	GG-
Wildtype	60	0	0	0	44	0	0	0	104	0	0	0
*mom-1(or10)*	1	5	0	3	8	6	0	2	9	11	0	5
*mom-1(se2)*	6	1	1	5	-	-	-	-	6	1	1	5
*mom-3(or78)*	11	5	2	7	10	11	1	19	21	16	3	26
*mom-5(RNAi)*	20	2	0	0	-	-	-	-	20	2	0	0
*mom-4(ne19)*	10	7	0	0	2	2	0	0	12	9	0	0
*mom-4(or39)*	14	7	0	0	-	-	-	-	14	7	0	0
*mom-4(or11)*	10	0	0	0	-	-	-	-	10	0	0	0

Number of embryos (N) expressing one of two E lineage-specific reporters: *Pend-1::gfp::h2b* (*end-1* reporter) or *Psdz-23::gfp::h2b* (*sdz-23* reporter). The *end-1* reporter was tested in wildtype (WT) embryos (N = 60), embryos mutant in the Wnt pathway: *mom-1*(or10), N = 9; *mom-1(se2)*, N = 13; *mom-3(or78)*, N = 25; *mom-5(RNAi)*, N = 22; or embryos mutant in the MAPK pathway: *mom-4(ne19)*, N = 17; *mom-4(or39)*, N = 21; *mom-4(or11)*, N = 10. The *sdz-23* reporter was tested in WT, N = 44; *mom-1(or10)*, N = 16; *mom-3(or78)*, N = 41; and *mom-4(ne19)*, N = 4.

Reporter expression results (GFP+/−) are correlated with gut granule development (GG+/−) for each individual embryo, generating 4 classes: GFP+/GG+, GFP−/GG+, GFP+/GG−, and GFP−/GG−.

### Discordant expression of three E-specific genes in P_2_-EMS signaling mutants

**Figure 2:**
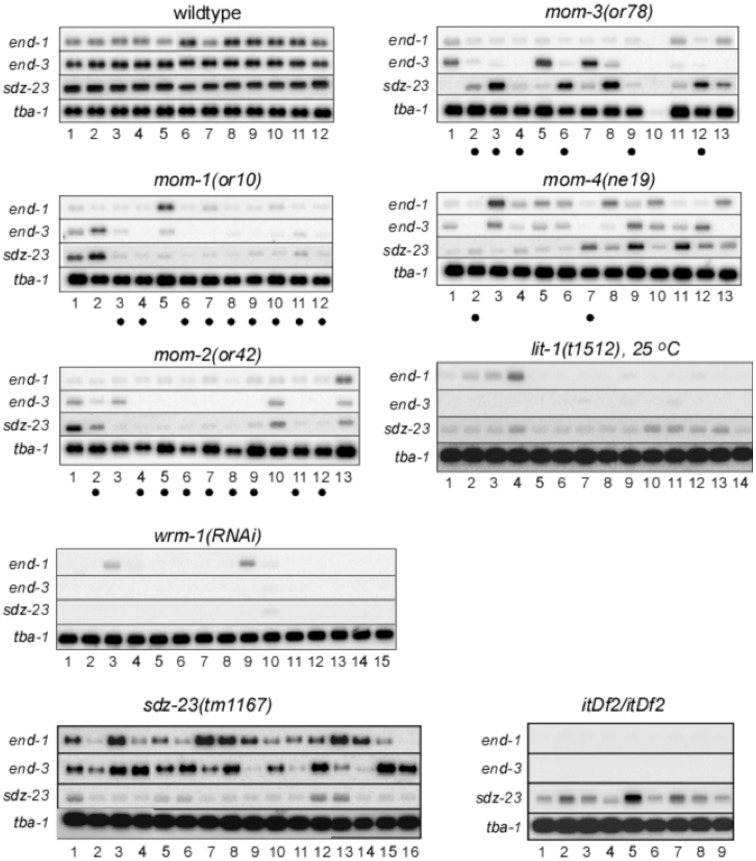
Single embryo gene expression analysis. Total cDNA was PCR amplified from poly-A+ RNA prepared from individual wildtype or mutant 12-cell stage embryos (indicated above each blot compilation), separated briefly on an agarose gel side-by-side with like-staged samples of the same genotype, and blotted to membrane ('pseudo Northern' blot; see Materials and Methods). Replicate blots were hybridized with ^32^-P labeled probes prepared from the *end-1*, *end-3*, and *sdz-23* genes, and the *tba-1* (alpha tubulin) gene as a loading control. Exposures were to x-ray film which was then scanned. All *end-1*, *end-3* and *sdz-23* exposures were for a similar length of time (18–20 hours), whereas the *tba-1* exposures were much shorter (10–15 minutes). *itDf2* is a deletion that removes the genomic region containing both *end-1* and *end-3* (along with a number of other genes). Dots below lane numbers denote samples from individual *mom-1(or10)*, *mom-2(or42)*, *mom-3(or78)* and *mom-4(ne19)* mutant embryos that express greatly reduced combined levels of *end-1* and *end-3*.

To examine the effect of P_2_-EMS signaling on multiple E-specific genes in the same embryo, we performed RT-PCR for individual precisely-staged wildtype or mutant embryos [38]. Total cDNA was prepared and amplified from individual 12-cell *C. elegans* embryos using the protocol of Brady [38], [39], as modified by us [37]. The quality of these total cDNAs was assayed by pseudo-Northern blot analyses as described [37]. As shown previously, we detected uniform expression of *end-1*, *end-3*, and *sdz-23* in all individual wildtype 12-cell embryos [37]. Examination of *end-1*, *end-3*, and *sdz-23* expression in individual 12-cell embryos from P_2_-EMS signaling mutants resulted in several novel insights into the regulation of these genes (Figure 2). First, the three E-specific genes were not affected in a similar fashion by the same P_2_-EMS mutation at the level of single embryos. This is most striking for individual 12-cell *mom-4(ne19)* and *mom-3(or78)* mutant embryos. Some embryos expressed a wildtype level of one E-specific gene and low levels or none of the other two genes (e.g. *mom-3(or78)*, lanes 3, 5, 6, 7, 8 and 12). Other embryos were defective in only one of the three E-specific genes (eg. *mom-1(or10)*, lane 2, and *mom-4(ne19)*, lanes 3 and 9). Second, although the number of embryos examined is small, we noticed that the overall number of embryos within a mutant group that exhibited reduced combined expression of the *end-1* and *end-3* genes roughly correlated with the number of embryos that typically fail to produce intestine for that mutation or RNAi treatment. The *mom-1(or10)*, *mom-2(or42)*, *mom-3(or78)* and *mom-4(or39)* mutations produce 85%, 72%, 65% and 40% of embryos lacking intestinal cells, respectively [22]. We observed 9 of 12 (75%), 9 of 13 (69%), 6 of 12 (50%), and 2 of 13 (15%) embryos with very reduced combined *end-1* and *end-3* expression among the *mom-1(or10)*, *mom-2(or42)*, *mom-3(or78)* and *mom-4(ne19)* mutant embryos (these samples are marked by dots below lane numbers in Figure 2). Close to one hundred percent of the embryos derived from *wrm-1(RNAi)* or *lit-1(t1512)* worms did not make intestine, and, accordingly, very low expression levels for all three genes was observed in most embryos.

We also analyzed by RT-PCR the expression of *end-1* and *end-3* in embryos deleted of *sdz-23* (*tm1167*), as well as the expression of *sdz-23* in embryos carrying a deficiency, *itDf2*, which completely removes both *end-1* and *end-3*, among other genes [8]. The *tm1167* mutation is a deletion of 428 basepairs at the *sdz-23* locus that removes 70 nucleotides upstream of the initiator AUG, as well as 358 nucleotides from the first exon, and is therefore likely to be a null mutation in *sdz-23*. We detected expression of *sdz-23* in *itDf2* homozygous embryos, and expression of *end-1* and *end-3* in *sdz-23(tm1167)* embryos (Figure 2). Although the expression of *sdz-23* in *itDf2* embryos and of *end-1/3* in *sdz-23(tm1167)* embryos are more variable than those detected in wildtype embryos in our assays, these results suggest that expression of *sdz-23* is not strictly dependent upon *end-1/3* expression, and vice versa.

All together, these results support a model whereby transcriptional activation of *end-1*, *end-3*, and *sdz-23* by the P_2_-EMS signal is regulated without a strict interdependence between the genes, and each gene responds individually to a reduction in the P_2_-EMS signal. Furthermore, expression of *end-1* and *end-3* appears to vary stochastically when individual embryos are compared, consistent with the combined expression levels of both *end-1* and *end-3*, rather than either gene individually, being critical for specifying E to differentiate intestinal markers.

The assays employed in measuring 1) expression of the E-lineage reporters from a multicopy transgene at single cell resolution, and 2) endogenous mRNA levels in single 12-cell embryos by RT-PCR, differ significantly in sensitivity and therefore cannot easily be directly compared. However, each assay is internally controlled, and the two assays do show a correlation. For example, in the *mom-3(or78)* sample sets, 6 of 15 embryos expressed no detectable amounts of the *end-1* promoter-driven transgene, and 6 of 15 embryos did not generate gut granules. The RT-PCR data showed variable and generally low, but not absent, levels of *end-1* expression (compare to the deletion mutant where both *end-1* and *end-3* genes are completely missing). Five of these 12 RT-PCR samples have a very low combined *end-1* and *end-3* expression (lanes 3, 4, 6, 9, 12), and therefore are unlikely, we would predict, to generate gut granules if allowed to develop.

### E lineage-specific *wee-1.1* expression is regulated by the P_2_-EMS signal

*wee-1.1* encodes the only zygotically-expressed WEE1-like kinase, a negative regulator of the cyclin-dependent kinase CDC2, and therefore a negative regulator of cell cycle progression [40]. Previous in situ hybridization showed that *wee-1.1* message is expressed very briefly in 12–16-cell embryos corresponding to a 10–15 minute developmental window. *wee-1.1* expression was only detected in the E blastomere and the 8 descendants from the AB blastomere. A reporter containing the upstream regulatory sequence of *wee-1.1* driving GFP expression was detected in both E daughters [36].

**Figure 3:**
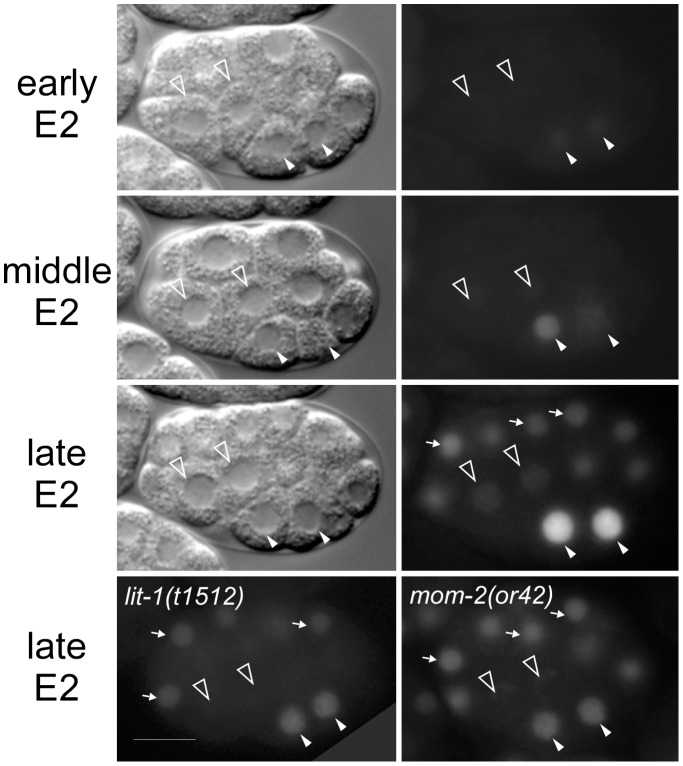
*wee-1.1* expression in the early embryo is Wnt/MAPK-dependent. Top three rows show three time points of the same embryos from early 2E to late 2E stage. DIC images (left-hand column) and fluorescence micrographs (right-hand column) of embryos expressing the *wee-1.1* reporter. The bottom row shows the expression of the same transgene in 2E-staged *lit-1(t1512)*, left, and *mom-2(or42)*, right, mutant embryos. Closed arrowheads indicate the 2E nuclei. Open arrowheads indicate the 2MS nuclei. Arrows point to AB nuclei expressing the reporter GFP. Note the reduced (but not absent) expression of the *wee-1.1* reporter in the 2E nuclei in the *lit-1* and *mom-2* mutants. Bar: 10 µm.

We investigated whether *wee-1.1* regulates the cell cycle and whether its expression in the E lineage is dependent on the P_2_-EMS interaction. We generated a GFP reporter that contains 1 kb of upstream sequence of the *wee-1.1* gene fused to *gfp::histone H2B*. The stable nature of GFP::H2B makes this GFP a valid reporter for the onset of *wee-1.1* transcription/translation, but not WEE-1.1 protein level. We detected high levels of GFP expression in the two E daughters immediately after E divides (Figure 3). A weaker GFP signal was also detected in daughters of the 8AB blastomeres soon after, consistent with the in situ data shown previously [40]. We found that the high nuclear GFP fluorescence of the *wee-1.1* reporter in the 2E cells is dependent on the P_2_-EMS interaction (Figure 3). In embryos homozygous for the *mom-2(or42)* Wnt ligand mutation, or for the MAPK temperature-sensitive mutation, *lit-1(t1512)*, elevated *wee-1.1* reporter GFP fluorescence in the 2E cells was not observed. The GFP fluorescence in the AB cells remains unchanged from wildtype in either mutant embryo, showing that the P_2_-EMS signal affects *wee-1.1* expression only in the E lineage. The *wee-1.1* GFP level in 2E cells in *mom-2(or42) and lit-1(t1512)* embryos is reduced to a level comparable to that detected in the AB lineage. This result indicates that the P_2_-EMS signal is not required for *wee-1*.1 basal expression, but is required for the high level expression observed specifically in the E lineage.

### *wee-1.1* negatively regulates the cell cycle in early embryos

Worms homozygous for *wee-1.1(ok418)*, which deletes 718 bp of the *wee-1.1* locus, including the translation initiation codon and the first half of the coding sequence, are viable and produce viable embryos with a normal-looking intestine. This suggests that the *wee-1.1* gene is not essential for viability, specification and differentiation of the intestine, or gastrulation and morphogenesis (data not shown).

**Figure 4:**
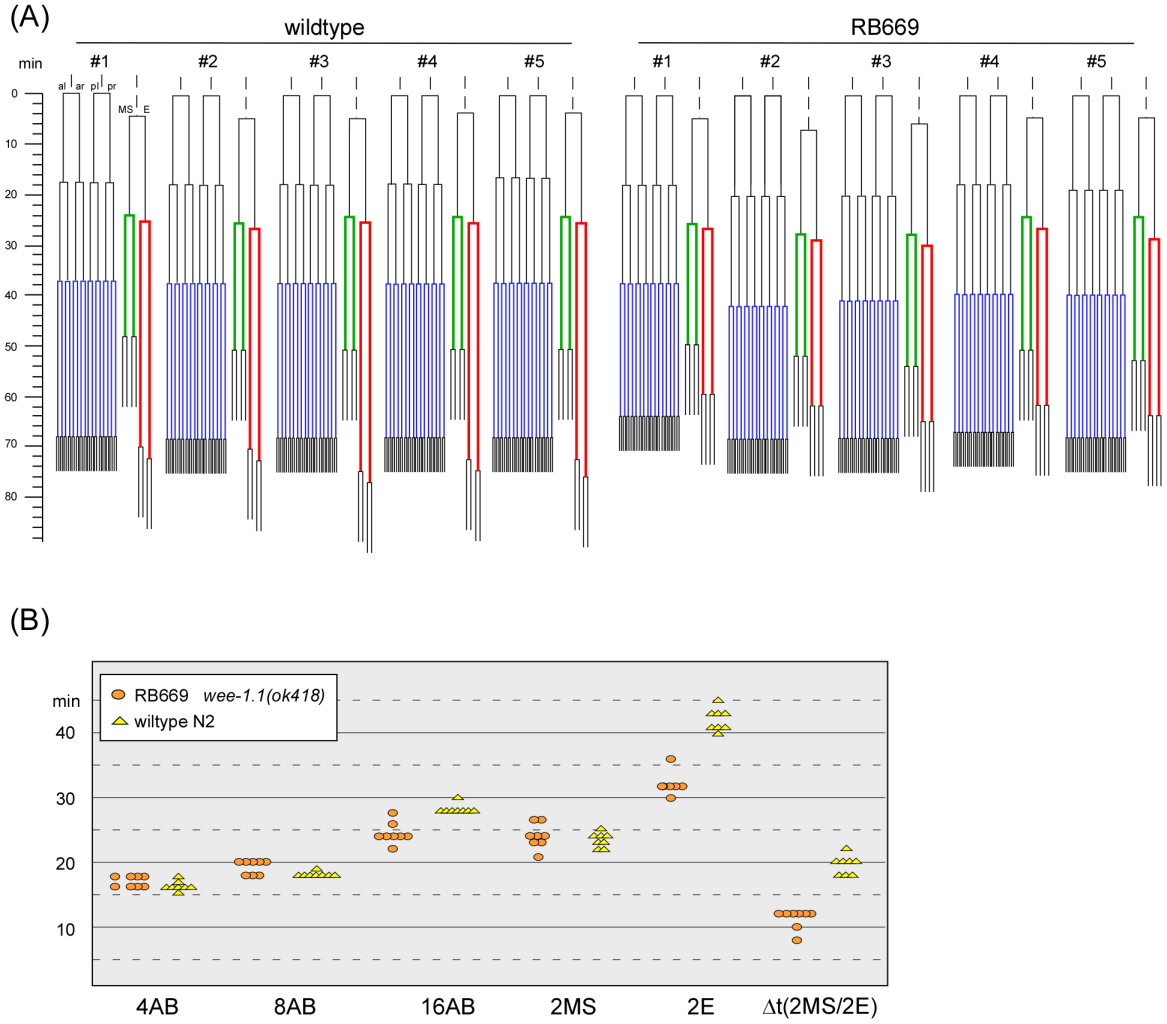
4D lineaging analyses of wildtype and *wee-1.1(ok418)* mutant embryos. (A) Lineaging results for the AB, MS and E lineages are shown for five wildtype embryos (left) and five *wee-1.1(ok418)* mutant embryos (strain RB669, right). The lineaging results are shown starting from the 3-cell stage to approximately the 50-cell stage. The 2AB to 4AB division is taken as time 0. The 16AB cell cycle is shown in blue, the 2MS cell cycle in green, and the 2E cell cycle in red. (B) Summary of specific cell cycle times for all wildtype and *wee-1.1(ok418)* mutant embryos examined.

To analyze whether cell division timings were altered in embryos lacking *wee-1.1*, we lineaged the embryonic cell divisions of 8 *wee-1.1(ok418)* and 8 wildtype embryos for the period from the 3-cell stage to approximately the 50-cell stage. The lineage of five embryos for each genotype is shown in Figure 4A, and cell cycle timings for all eight embryos for each genotype are plotted in Figure 4B. We detected no abnormality in cell division pattern in *wee-1.1 (ok418)* embryos versus wildtype embryos until the 28-cell stage when the 2E cells (Ea and Ep) divide. In wildtype embryos, the 2E division occurs approximately 19 minutes (n = 8, SD = 1 min.) after the 2MS division (Figure 4B). In *wee-1.1(ok418)* embryos, we observed that the 2E division occurs only 10 minutes (n = 8, SD = 1 min.) after the 2MS division. This difference resulted from a shortened 2E cell cycle of 32 minutes (SD = 2 min.) in *wee-1.1 (ok418)* embryos, compared to 42 minutes (SD = 2 min.) in wildtype embryos. No differences were observed for the 2MS cell cycles between wildtype and *wee-1.1 (ok418)* embryos [23 minutes (SD = 1 min.) and 24 minutes (SD = 1 min.), respectively].

Consistent with the reported *wee-1.1* expression in the 8AB blastomeres in wildtype embryos, we also observed a slight reduction in the 16AB cell cycle duration, from an average of 28 minutes (SD = 1 min.) in wildtype embryos to 24 minutes (SD = 2 mins.) in *wee-1.1(ok418)* mutant embryos. This effect is much less than the effect observed for the 2E cell cycle, but nonetheless was consistent and significant.

**Figure 5:**
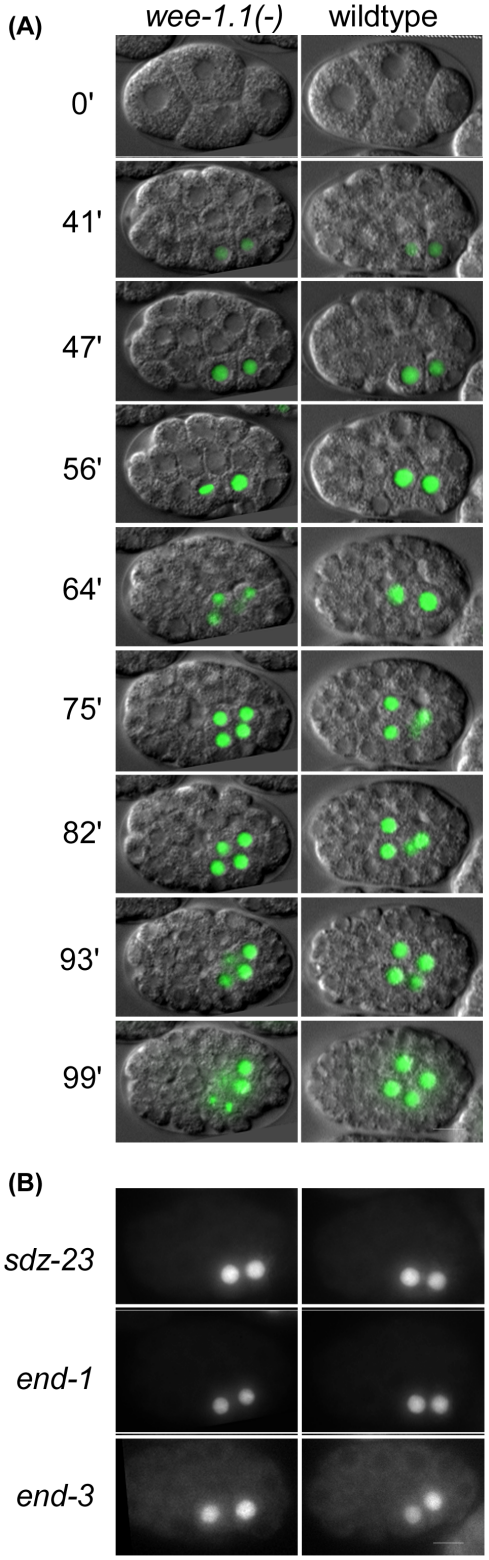
E lineage reporter expression in wildtype versus *wee-1.1(ok418)* embryos. A. Time course showing merged DIC and nuclear *end-1* reporter GFP in a *wee-1.1(ok418)* (left) and wildtype (right) live embryo. 4-cell stage taken as time 0. Reporter expression is shown at eight different timepoints from 41 minutes (2E) until 99 minutes (4E stage). Note that the 2E cells in the *wee-1.1* mutant embryo initiate mitosis at t = 56, 10 minutes before the wildtype 2E cells initiate mitosis. B. All three E-lineage-specific reporters exhibit wildtype expression in *wee-1.1(ok418)* mutant embryos (2E stage shown). Bar: 10 µm.

We also performed 4-D imaging of *wee-1.1(ok418)* embryos expressing the E lineage-specific *end-1*, *end-3*, or *sdz-23* reporters. We found the expression levels of *end-1*, *end-3*, and *sdz-23* reporters to be indistinguishable between wildtype and *wee-1.1(ok418)* mutant embryos (Figure 5). Figure 5A shows comparable stages of wildtype and *wee-1.1(ok418)* mutant embryos from 4-D recordings in which the DIC images were overlaid with the corresponding *end-1* reporter GFP expression.

To determine whether *wee-1.1(ok418)* embryos exhibited any defects in the number of E lineage cells generated, we allowed mutant and wildtype embryos expressing the E-lineage specific reporters to develop until shortly before hatching. These two groups of late-stage embryos were stained with anti-GFP antibody and the number of GFP-positive cells counted. The cell counts centered on 20 with little variance in both the wildtype and the *wee-1.1* mutant embryos (n = 10 for both groups, data not shown). We conclude that *wee-1.1* regulates the cell cycle duration of a part of the E lineage, but does not function as a counter of cell division number.

Together, our analysis of *wee-1.1(ok418)* mutant embryos demonstrates that *wee-1.1* negatively regulates cell cycle duration in early *C. elegans* embryos, and its high level expression in E accounts, at least in part, for the long cell cycle duration of Ea and Ep. P_2_-EMS signaling regulates the 2E cell cycle duration by promoting the high level transcription of *wee-1.1* in the E blastomere. We found no indication that *wee.1.1* regulates any of the other properties of the E lineage. We conclude that the stereotypic E lineage cell cycle control can be uncoupled from the specification of E as the intestinal precursor, and is not essential for gastrulation or embryogenesis.

### Uncoupling cell division number in the E lineage from differentiation of intestine

**Figure 6:**
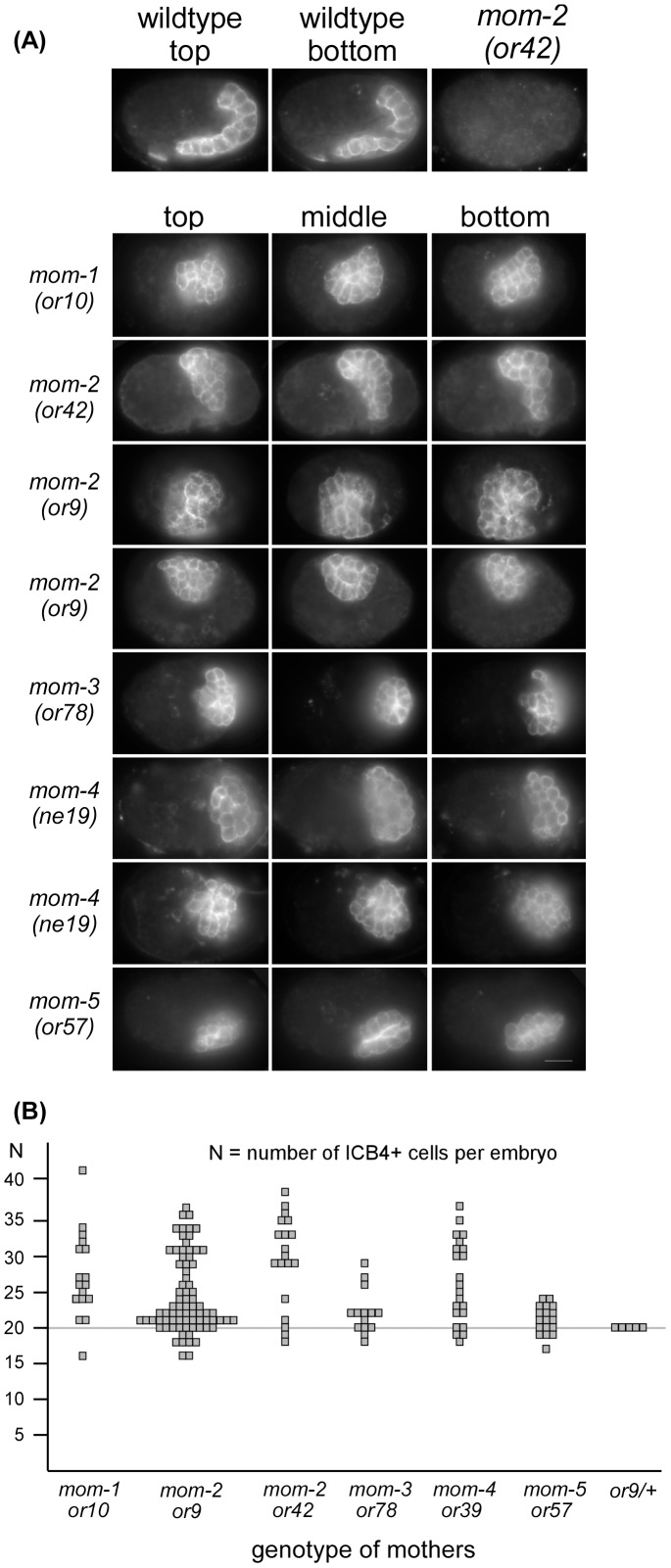
Intestine development in Wnt/MAPK mutant embryos. (A) Differentiated intestinal cells stained with ICB4 antibody. The first two panels of the top row show a wildtype embryo imaged at two different focal planes. The third panel is a representative *mom-2(or42)* mutant embryo that did not develop intestinal cells. The following rows are examples of embryos with the indicated mutation that did develop ICB4-positive cells, imaged at three different focal planes (top, middle, and bottom). Note that in all the mutant embryos the number of ICB4-positive cells exceeds 20. Furthermore, the intestine is not elongated as in the wildtype, and instead appears as a clump of small cells. (B) Summary of ICB4-positive cell counts per embryo for the indicated Wnt and MAPK mutants. Note that the number spread from the wildtype number (20) is much restricted for the Wnt frizzled receptor mutant, *mom-5(or57)*, versus the other mutants, and that *mom-2(or9/+)* heterozygous embryos develop the wildtype number of intestinal cells (indicating that a single wildtype copy of *mom-2* is sufficient to determine E lineage cell-division number).

We next asked whether the counting of cell division number could also be uncoupled from the differentiation of intestine by determining the number of intestinal cells in embryos carrying mutations affecting the P_2_-EMS signaling, a subset of which produces intestinal cells [20], [22]. We performed immunofluorescence using a monoclonal antibody against ICB4, an antigen expressed on differentiated gut cells [41]. Images of embryos were collected as 3-D stacks and the number of ICB4-positive cells were counted. In wildtype embryos, the ICB4 staining outlines the 20 intestinal cells, as well as the two intestinal-rectum valve cells [42], and these cells can be easily counted at an embryonic stage approximately 400 to 440 minutes after the 2-cell embryo (Figure 6A). We avoided counting embryos older than 440 minutes, which is when the intestinal cells start to elongate and the intestinal lumen starts to form. The 20 intestinal cells and 2 valve cells could be clearly counted in all wildtype embryos examined (100%, n = 50) (Figure 6B).

We observed that 77% and 72% of embryos derived from *mom-2(or9)* or *mom-2(or42)* homozygous mothers, respectively, produce no intestinal cells and therefore contain no or very few ICB4-positive cells [22]. We focused therefore on the other 23% and 28%, respectively, of the *mom-2* mutant embryos that did produce ICB4-positive cells. Because intestinal cells do not undergo proper morphogenesis in *mom-2* mutant embryos, we were not able to unambiguously identify the two intestinal-rectum valve cells, and therefore counted all ICB4 positive cells. Strikingly, among the *mom-2* mutant embryos that generated ICB4-positive cells, many embryos were found that had more than 20 ICB4-positive cells (Figure 6).

However, because of the low expression level of E markers in *mom-2* mutant embryos (see Figure 1 and Table 1) and the small percentage of mutant embryos that generated intestinal cells, we were unable to determine unambiguously the lineage source for the extra intestinal cells in the *mom-2* mutant embryos. Nevertheless, we believe that the extra intestinal cells resulted from extra rounds of cell division within the E lineage for the following three reasons. First, when imaging early *mom-2* mutant embryos expressing the E lineage reporters with the *end-1*, *end-3*, or *sdz-23* promoters driving GFP::H2B, we never observed GFP signal in any cells outside of the E lineage (n>200). Second, ELT-2 is an E-lineage-specific GATA factor which begins to be expressed in the wildtype embryo at the 4E stage. Using a reporter that expressed ELT-2::GFP under the control of the *elt-2* promoter, we followed the number of GFP-positive cells from the 4E stage in the subset of *mom-2* mutant embryos that generated intestine (n = 24). We observed 5 embryos that produced more than 29 ELT-2-positive cells before they stopped dividing due to UV-induced damage (Figure 6B). In one of these embryos, we counted a minimum of 32 ELT-2::GFP-positive cells. Third, in those *mom-2* mutant embryos exhibiting more than 20 ICB4-positive cells, these cells tended to be smaller than the individual ICB4-positive cells of similarly staged wildtype embryos. This characteristic small size was also associated with the supernumerary intestinal cells that are generated in *cdc-25(gof)* and *cki-1(-)* mutant embryos [33], [35]. We conclude that our results are consistent with the extra gut cells in *mom-2* mutant embryos deriving from extra divisions within the E lineage. This suggests that the P_2_-EMS signal, and specifically the Wnt pathway, normally restricts the total number of E lineage cell divisions.

We also observed more than 20 ICB4-positive cells in embryos mutant for other genes in the Wnt signaling pathway, including mutations in *mom-1* (Porcupine) and *mom-3* (Wntless), as well as for the gene encoding the MAP3K *mom-4* (Figure 6). Only a very slight increase in ICB4-positive cells was observed in *mom-5* (Frizzled) mutant embryos. This low penetrance defect correlates with the low penetrance defect in intestinal formation (5%) for all *mom-5* mutants, suggesting that depletion of Frizzled by itself does not abolish the downstream signaling pathway readout. Altogether, our results suggest that the P_2_-to-EMS signal, in addition to specifying E as the endodermal precursor, also restricts the number of cell divisions in the E lineage.

## Discussion

An E blastomere isolated from an 8-cell embryo exhibits many properties of the E lineage in an intact embryo. Here we show that the same genes that function during P_2_-to-EMS signaling to specify E to make intestinal cells also regulate several other properties of the E lineage, including the Ea and Ep extended cell cycle as well as the total number of cell divisions in the E lineage. P_2_-EMS signaling activates high level expression of a negative cell cycle regulator, *wee-1.1*, in the E blastomere, and extends the Ea and Ep cell cycle duration. Our observations suggest that the interaction in the 4-cell embryo between P_2_ and EMS not only activates genes required for differentiation of intestinal cells, but it also specifies most of the E lineage developmental program. While these E properties are determined by the interaction that occurred between P_2_ and EMS, they are affected differently in mutant embryos defective in this interaction and can be uncoupled. In addition, for three E-specific genes that are each expressed uniformly between wildtype embryos, we detected tremendous variability in expression levels as well as a distinct discordance in expression between the three genes in individual embryos mutant for P_2_-EMS signaling components. These results illustrate a lack of strict dependence between transcriptional activation of these genes, consistent with these three E lineage-specific genes not being regulated in a simple linear pathway. *end-1* expression appears to be primarily regulated via the P_2_-EMS interaction and POP-1, *end-3* expression primarily through the SKN-1 and MED-1/2 pathway, and *sdz-23* expression, although dependent upon the P_2_-EMS signal, appears to be at least partly independent of *end-1/3* expression.

**Figure 7:**
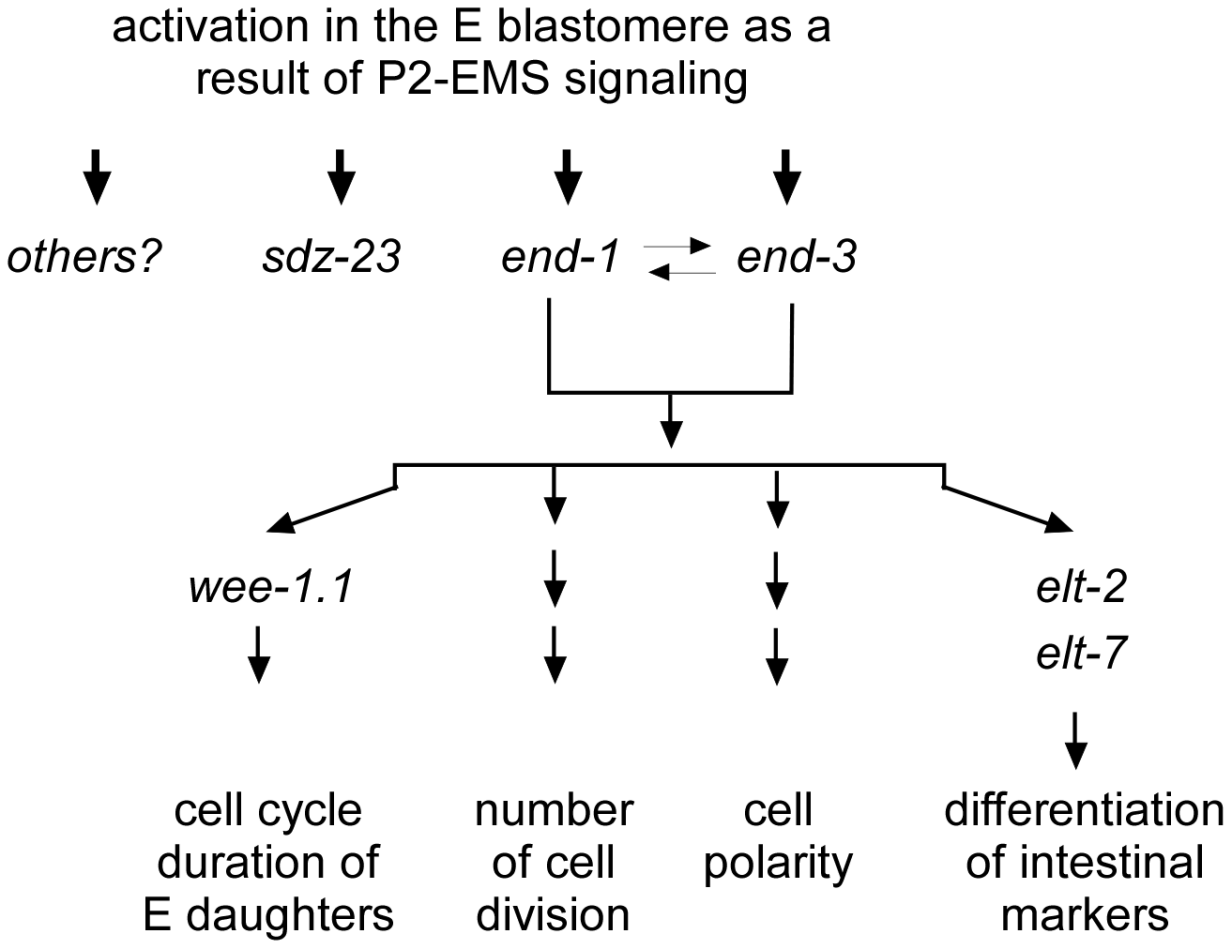
Model. *end-1* and *end-3* are activated in the E blastomere as a result of P_2_-to-EMS signaling and function redundantly to regulate all currently quantifiable characteristics of the E lineage. However, there are also genes, like *sdz-23*, that are expressed specifically in the E lineage and which also require P_2_-to-EMS signaling for their activation. Intriguingly, these genes do not appear to be involved in intestine specification or development, and do not appear to be regulated by *end-1* or *end-3*.

There are two likely models whereby the P_2_-EMS interaction at the 4-cell stage regulates the many properties associated with the E lineage. One possibility is that the P_2_-EMS interaction results in the direct activation of a suite of genes, each of which regulates separate pathways that together lead to the full E lineage phenotype. A second possibility is that the P_2_-EMS interaction activates the expression of a master gene in E, which then activates the expression of a number of downstream target genes, resulting in the generation of the complete E phenotype. Much of our current results favor the second model. While not all embryos that generate intestinal granules display all characteristics of the E lineage, embryos that do not produce intestinal granules exhibit none of the E lineage characteristics [35], [43]. In the second model, the *end-1* and *end-3* gene pair would be a likely candidate for such a master gene. *end-1* is a direct target of the TCF transcription factor POP-1 [23]. It has been shown that ectopic expression of *end-1* in a non-E lineage is sufficient to turn on the transcription of intestinal differentiation markers through a transcription cascade similar to that in the E lineage [6], [9]. Although there is no evidence that *end-3* is a direct target of TCF/POP-1 (Figure S1 and S2), *end-3* functions redundantly with *end-1* in embryos to regulate intestine development, and ectopic expression of *end-3* alone is also sufficient to activate transcription of intestinal markers in early embryos [6]. In embryos deleted of the chromosomal region including *end-1* and *end-3*, or precise *end-1(-)*; *end-3(-)* double mutants, E does not make intestine, nor does it exhibit any other properties characteristic of a wildtype E [8], [44]. These observations support the model that combined high level expression of *end-1* and *end-3*, resulting from the P_2_-EMS signal as well as input from the *skn-1* and *med-1/2* genes, activates a suite of downstream genes, each regulating different properties of a wildtype E lineage (Figure 7). It is likely therefore that *end-1* and *end-3* activity lead to the elevated expression of *wee-1.1* in Ea and Ep, as well as activation of an E-lineage cell cycle regulator that controls, many hours later, the total number of E-lineage cell divisions. However, our results for *sdz-23* demonstrate that the P_2_-to-EMS signal can also generate activation of an immediate target gene that appears to be independent of the *end-1* and *end-3* genes. Therefore, although the P2-to-EMS signal functions primarily through *end-1* and *end-3* to specify the E blastomere as the intestine founder cell through activation of downstream genes such as *elt-2* and *elt-7* [45], we find that many of the known E lineage characteristics can be uncoupled from intestinal cell differentiation.

The discordance we observe for defects in differentiation of intestinal markers, E2 cell cycle duration, and the total number of cell divisions in the E lineage, likely reflects a difference in sensitivity of each downstream process to the reduction of P2-EMS signaling. Our results suggest, for example, that the mechanism by which the E lineage counts cell division numbers is more sensitive than differentiation of intestinal markers to a reduction of P2-EMS signaling. Mutations in genes in the Wnt signaling pathway also have defects in gastrulation. Similar discordance between intestinal marker production and gastrulation was also observed in individual mutant embryos [43]. However, as it is difficult to assay gastrulation in the isolated E blastomere, it remains unclear whether Wnt signaling regulates gastrulation through the P2-EMS interaction or a different interaction at a later stage. Knockdown of the *sdz-23* gene by RNAi gave a mild gastrulation defect in a sensitized background, suggesting it may, directly or indirectly, regulate gastrulation [46].

Expression of *end-1*, *end-3*, and *sdz-23* appears to require direct binding of GATA transcription factors MED-1 and MED-2, which are active in both MS and E blastomeres [35]. Wnt-regulated TCF/POP-1 activity determines high level expression in E and repression in MS of these three E-specific zygotic genes [22]. *end-1* has been shown to be a direct target of POP-1 [22]. It is not clear how the Wnt signal results in the activation of *sdz-23* or *end-3*. Mutating all three putative TCF/POP-1 binding sites on the *end-3* upstream sequence did not result in any detectable abnormality in expression of an *end-3* reporter (Figure S1 and S2). *sdz-23* contains no putative TCF/POP-1 binding sites within a genomic fragment capable of regulating E lineage-specific expression. While *end-3* can activate *end-1* [13], and, to a smaller degree, *end-1* can activate *end-3* (our unpublished results), two observations argue against any simple linear regulatory relationship between these three genes. First, expression of *sdz-23* in the *itDf2* deletion and expression levels of *end-1* plus *end-3* in *sdz-23(tm1167)* mutant embryos are not grossly disrupted. Second, heatshock expression of *end-1* does not result in elevated expression of *sdz-23* (S. R and R. L., unpublished result). Our RT-PCR analyses showed discordance in the expression of *end-1*, *end-3*, and *sdz-23* in individual embryos carrying mutations in genes of the P_2_-EMS signal pathways. This discordance is more consistent with a model whereby the P_2_-EMS signal (and hence POP-1) regulates these three target genes in such a way that they are relatively independent of one another and do not function in a simple linear pathway. The observation that the three targets are not affected consistently from embryo to embryo carrying the same mutation suggests that the effect of a reduction of Wnt signaling to the expression of each target gene is, to some extent, a stochastic event. Similar stochastic defects in gene expression were also observed in embryos mutant for other transcription factors [7]. However, if the P_2_-EMS signals directly activate *end-3* and *sdz-23* transcription through TCF/POP-1 binding to sequences upstream of these genes, it would suggest that an atypical TCF/POP-1 binding sequence may be involved. We suggest that the P_2_-EMS signaling regulates these three target genes independently (Figure 7). The combined expression of *end-1* and *end-3* drives the known E lineage developmental programs. Our results concerning *sdz-23*, which does not appear to function in differentiation of intestine, raise the intriguing possibility that the P_2_-EMS interaction has downstream molecular consequences within the E blastomere, and the E lineage, in addition to its specification as the intestinal precursor. Continuing studies on function of *sdz-23* and other E lineage restricted genes will likely lead to the discovery of developmental programs operating in the E lineage that regulate more than intestine development.

## Materials and Methods

### Strains

N2 was used as the wildtype strain. Genetic markers: LGI, *dpy-5(e61)*, *mom-5(or57)*, *mom-4(ne19)*, *mom-4(or39), hT1(I:V), szT1(I:X)*; LGII, *wee-1.1(ok418)*, *rol-1(e91), mom-3(or78), mnC1*; LGIII, *unc-119(ed3)*, *lit-1(t1512)*, *unc32(e189)*; LGIV, *sdz-23(tm1167)*; LGV, *mom-2(or42), mom-2(ok591), itDf2, DnT1(IV;V)*; LGX, *mom-1(or10), mom-1(se2), unc-6(n102)*. MR156 (*rrIs01* [*elt-2::GFP; unc-119(+)*]) (gift from R. Roy [35]), TX895 [*unc-119(ed3)III; him-3(e1147)IV*; *teIs84(Pend-3::GFP::H2B)X*], TX585 [*unc-119(ed3)III; teIs18*(*P_sdz-23_gfp::H2B*)(*V*)] [23], TX691 [*unc-119(ed3)III; teIs46*(*P_end-1_gfp::H2B*)(*IV*)] [23], TX1499 [*unc-119(ed3)III; teEx696(P_wee-1.1_ gfp::H2B)*]. *P_wee-1.1_ gfp::H2B* was generated by recombining the 1 kb genomic sequence upstream of *wee-1.1* into pRL1075 using Gateway technology (Invitrogen) as described [37]. All other strains were obtained from the *Caenorhabditis* Genetics Center (CGC).

### RNA interference

RNAi was performed by feeding L3 larvae with RNAi bacteria according to Timmons et al [47]. Embryos laid 48-60 hours after feeding were collected and processed for cDNA isolation.

### Analysis of embryos and imaging

Expression of E-specific genes in wildtype and mutant embryos were analyzed as described previously [23]. Stage-matched embryos were collected from mutant and wildtype hermaphrodites, respectively, imaged side-by-side for GFP fluorescence, and then scored later for intestinal differentiation. Intestinal cells were identified by their birefringent gut-specific granules under polarized optics.

Live fluorescence images of E-specific GFP reporters were also collected as fluorescence and matching DIC stacks along the Z-axis as described [24], [48], [49].

Embryos were prepared for immunofluorescence using the anti-ICB4 antibody as described [41]. Images were collected as stacks along the Z-axis and the numbers of ICB4 positive cells were counted.

### Lineaging

Cell lineage analysis [1] was performed on wildtype N2 and *wee-1.1(ok418)* by four-dimensional time-lapse analysis. Differential interference contrast (DIC) images were collected at two-minute intervals from the 2-4-cell stage to the 4E stage.

### Single embryo expression analysis

The single embryo gene expression analyses were performed with *C. elegans* embryos as described [37]. The PCR amplified total cDNA is derived from total poly-A+ mRNA. Equal amounts of cDNA from individual embryos of an identical genotype were replica loaded onto agarose gels, electrophoresed until the bromophenol blue marker had moved approximately 3 cm into the gel, transferred to GeneScreen Plus membrane, and then hybridized with the labeled probes.

## Supporting Information

Figure S1 (A) Schematics of the genomic region upstream of the initiating AUG for both *end-1* and *end-3*. Blue triangles mark putative MED-1/2-binding sites (see below). Solid red and open red triangles mark putative POP-1 binding sites with, or without, associated Helper binding sequences, respectively (see below). The putative POP-1 binding sites begin at −171 and −1,027 for *end-1*, and at −139, −1,060, and −1,117 for *end-3*. Numbering is with respect to the putative AUG translation initiation codon (Uppercase/bold/green text, A = +1). Coding sequence is highlighted in yellow. Single arrowheads indicate orientation of the binding site relative to the consensus sequence (see below). Bold red underlined text indicates match to the consensus sequence. Triple arrowheads indicate protein and direction of translation. (B) GFP fluorescence micrographs of 4EMS stage embryos expressing a reporter containing a 1.7 kb of the *end-1* upstream sequence with the putative POP-1 site at −146 mutated (top panel), or a reporter containing 1270 bp of the *end-3* upstream sequence with all three putative POP-1 sites mutated (bottom panel). Note that expression of the *end-1* reporter with the most proximal POP-1 binding site mutated is both reduced in E lineage cells and derepressed in MS lineage cells. Compare that to expression of the *end-3* reporter with all three putative POP-1 binding sites mutated, which appears wildtype (i.e. high expression in the E lineage and no (repressed) expression in the MS lineage.
(TIF)

Figure S2 Upstream regulatory sequences for the *end-1*, *end-3* and *sdz-23* genes, highlighting the location of putative SKN-1 (ATTGTCAT) [1], MED-1/2* (RRRAGTATAC, green highlight) [50], POP-1 (GWWCAAAG, blue highlight) [51], and POP-1 Helper (GCCRVNW, blue highlight) [52] binding sites. R = G or A; W = A or T; V = G, C or A; N = G, T, A or C. Possible binding sites upstream of the *sdz-23* gene are highlighted in gray. POP-1 Helper sites are bound by the C-clamp domain immediately C-terminal of the HMG DNA-binding domain of TCF proteins [53] and are required for activation of Wnt target genes in E [54], [52]. Helper binding sites are usually found located on both sides of the consensus TCF binding site. Note that *end-1*, which is primarily activated in E by POP-1 binding [23] has a strong POP-1 activator site between 2 closely flanking MED-1/2 binding sites within 206 bp of the putative AUG initiator codon. The *end-1* upstream regulatory sequences have all the earmarks of a predominantly POP-1 activated gene in E. *end-3* also has, within 155 bp upstream of the initiator AUG, two MED-1/2 sites with a putative POP-1 binding site between them, although the POP-1 Helper sequences give a slightly poorer match to the consensus. Interestingly, in this case the distal POP-1 Helper sequence overlaps with the immediately upstream MED-1/2 binding site (the three overlapping nucleotides are shown in blue). In addition, there are 2 additional MED-1/2 binding sites within an additional 87 bp upstream. There is also a putative POP-1 consensus binding site, lacking apparent Helper sequences, a little over 800 bp further upstream from the most proximal MED-1/2 binding site. The *end-3* upstream regulatory sequences are certainly consistent with the evidence showing significant *end-3* regulation via MED-1/2 binding. The POP-1 site including flanking Helper binding sequences would also suggest a role for POP-1 in *end-3* activation in E, but our mutational analyses to date do not support this (Figure S1). The *sdz-23* upstream regulatory sequence shows one MED-1/2 site (and probably a second) within 215 bp of the initiator AUG, as well as a possible SKN-1 binding site 117 bp further upstream. How *sdz-23* is transcriptionally activated as a result of the P2-EMS Wnt signal is currently unknown. *The MED-1 and MED-2 DNA binding domains (GATA-factor single zinc fingers) differ by only 1 amino acid, and therefore it is highly likely that they bind the same DNA sequence.
(TIF)
