# Supplementary figures and images for: Uncoupling Different Characteristics of the C. elegans E Lineage from Differentiation of Intestinal Markers
Source: PLoS One. 2014 Sep 2;9(9):e106309. doi: 10.1371/journal.pone.0106309 (PMC4152275; doi:10.1371/journal.pone.0106309)

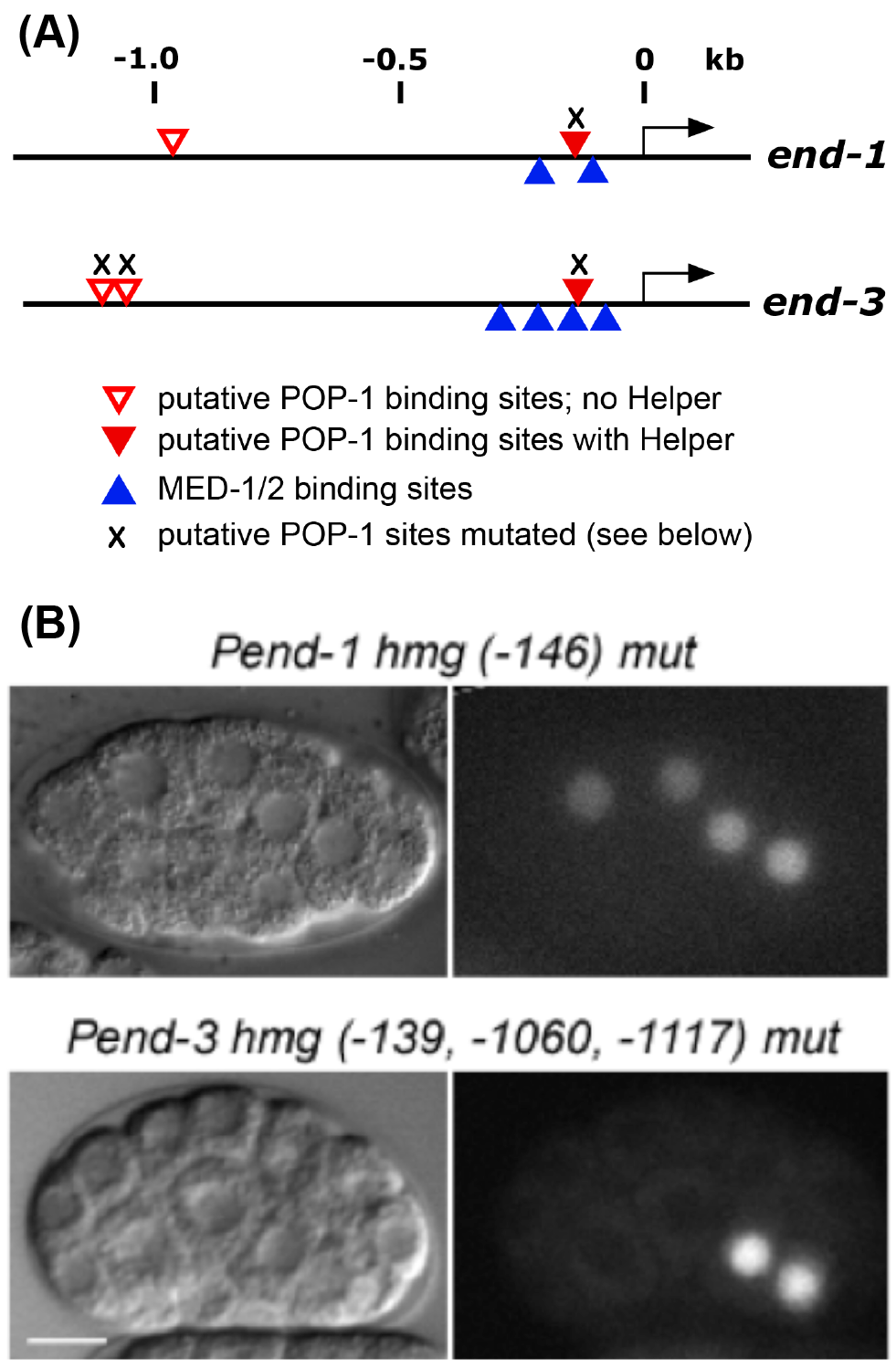

Supplement: Figure S1 — (A) Schematics of the genomic region upstream of the initiating AUG for both end-1 and end-3. Blue triangles mark putative MED-1/2-binding sites (see below). Solid red and open red triangles mark putative POP-1 binding sites with, or without, associated Helper binding sequences, respectively (see below). The putative POP-1 binding sites begin at −171 and −1,027 for end-1, and at −139, −1,060, and −1,117 for end-3. Numbering is with respect to the putative AUG translation initiation codon (Uppercase/bold/green text, A = +1). Coding sequence is highlighted in yellow. Single arrowheads indicate orientation of the binding site relative to the consensus sequence (see below). Bold red underlined text indicates match to the consensus sequence. Triple arrowheads indicate protein and direction of translation. (B) GFP fluorescence micrographs of 4EMS stage embryos expressing a reporter containing a 1.7 kb of the end-1 upstream sequence with the putative POP-1 site at −146 mutated (top panel), or a reporter containing 1270 bp of the end-3 upstream sequence with all three putative POP-1 sites mutated (bottom panel). Note that expression of the end-1 reporter with the most proximal POP-1 binding site mutated is both reduced in E lineage cells and derepressed in MS lineage cells. Compare that to expression of the end-3 reporter with all three putative POP-1 binding sites mutated, which appears wildtype (i.e. high expression in the E lineage and no (repressed) expression in the MS lineage. (TIF) [file pone.0106309.s001.tif]

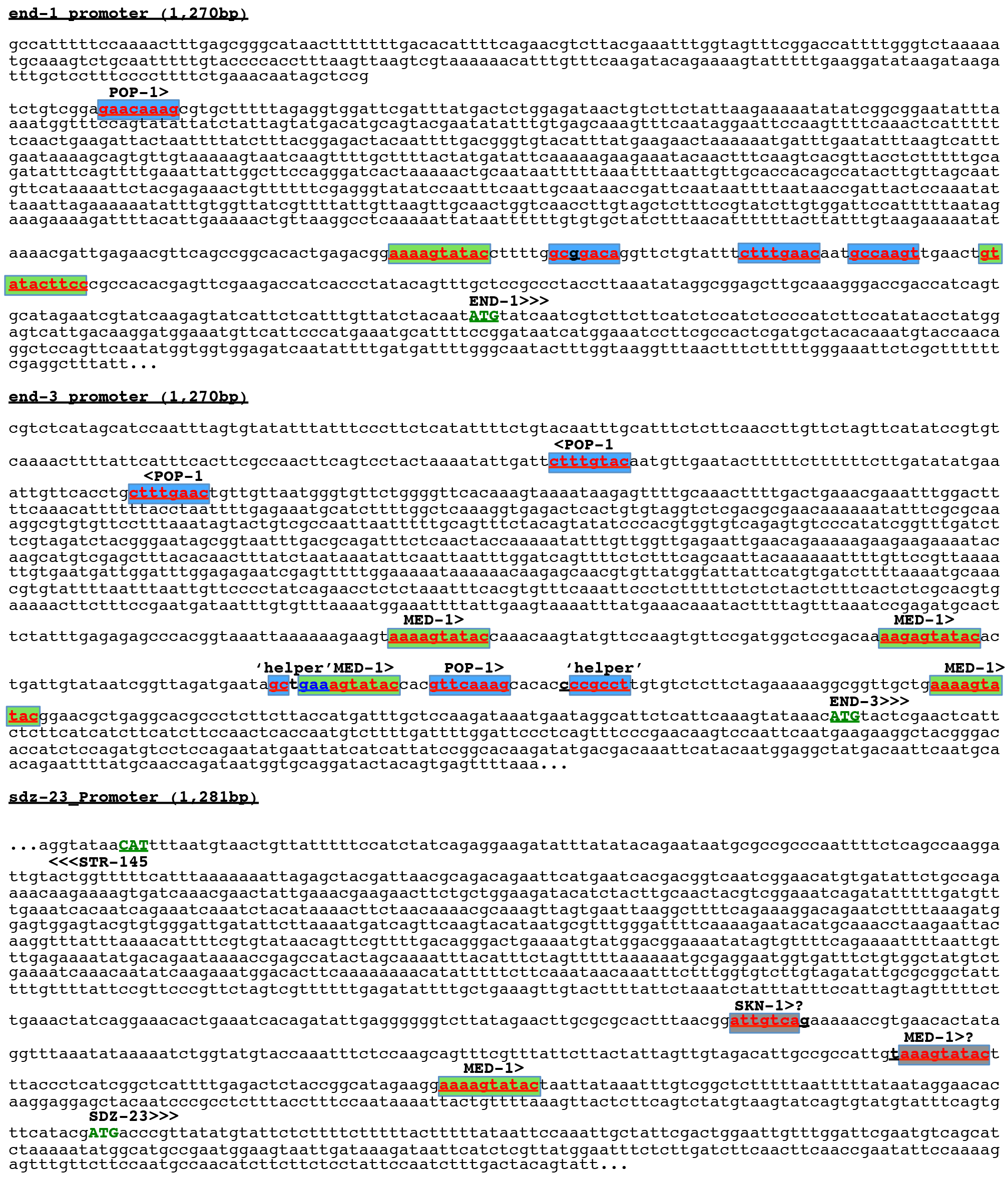

Supplement: Figure S2 — Upstream regulatory sequences for the end-1, end-3 and sdz-23 genes, highlighting the location of putative SKN-1 (ATTGTCAT) [1], MED-1/2* (RRRAGTATAC, green highlight) [50], POP-1 (GWWCAAAG, blue highlight) [51], and POP-1 Helper (GCCRVNW, blue highlight) [52] binding sites. R = G or A; W = A or T; V = G, C or A; N = G, T, A or C. Possible binding sites upstream of the sdz-23 gene are highlighted in gray. POP-1 Helper sites are bound by the C-clamp domain immediately C-terminal of the HMG DNA-binding domain of TCF proteins [53] and are required for activation of Wnt target genes in E [54], [52]. Helper binding sites are usually found located on both sides of the consensus TCF binding site. Note that end-1, which is primarily activated in E by POP-1 binding [23] has a strong POP-1 activator site between 2 closely flanking MED-1/2 binding sites within 206 bp of the putative AUG initiator codon. The end-1 upstream regulatory sequences have all the earmarks of a predominantly POP-1 activated gene in E. end-3 also has, within 155 bp upstream of the initiator AUG, two MED-1/2 sites with a putative POP-1 binding site between them, although the POP-1 Helper sequences give a slightly poorer match to the consensus. Interestingly, in this case the distal POP-1 Helper sequence overlaps with the immediately upstream MED-1/2 binding site (the three overlapping nucleotides are shown in blue). In addition, there are 2 additional MED-1/2 binding sites within an additional 87 bp upstream. There is also a putative POP-1 consensus binding site, lacking apparent Helper sequences, a little over 800 bp further upstream from the most proximal MED-1/2 binding site. The end-3 upstream regulatory sequences are certainly consistent with the evidence showing significant end-3 regulation via MED-1/2 binding. The POP-1 site including flanking Helper binding sequences would also suggest a role for POP-1 in end-3 activation in E, but our mutational analyses to date do not support this (Figure S1). [file pone.0106309.s002.tif]
